# Construction and validation of nomograms for predicting overall survival and cause-specific survival in cervical cancer patients undergoing radical radiotherapy based on the SEER database

**DOI:** 10.3389/fmed.2025.1587465

**Published:** 2025-04-29

**Authors:** Yadong Zhang, Liyi Rong, Hairong Jiang, Xin Mu, Hongfu Zhao

**Affiliations:** ^1^Department of Radiation Oncology, China-Japan Union Hospital of Jilin University, Changchun, China; ^2^Department of Geriatrics, Jilin City Hospital of Chemical Industry, Jilin City, China; ^3^Department of Radiation Oncology, Jilin City Hospital of Chemical Industry, Jilin City, China

**Keywords:** cervical cancer, SEER database, overall survival, cause-specific survival, radical radiotherapy

## Abstract

**Objective:**

This study aimed to construct and validate competitive-risk model nomograms using the Surveillance, Epidemiology, and End Results (SEER) database to predict the overall survival (OS) and cause-specific survival (CSS) for cervical cancer patients treated with radical radiotherapy from 2000 to 2020.

**Materials:**

Cervical cancer cases treated with radical radiotherapy in the SEER database were retrieved. Inclusion criteria included the pathological diagnosis of cervical cancer, diagnosis within the specified time frame, and age between 20 and 79 years. Exclusion criteria were the presence of other malignant tumors, cancer-directed surgery, and incomplete data. Variables such as age, diagnosis year, and race were extracted. Patients were randomly divided into a training set and a validation set at a 4:1 ratio using a stratified random sampling method. For the training set patients, univariate and multivariate Cox regression analyses were performed, and CRM nomograms to predict the 3-year and 5-year OS and CSS were created in R. Harrell’s concordance index (C-index) and a calibration curve were used to assess model performance.

**Results:**

A total of 8,810 patients were included for OS and CSS analysis. The median follow-up was 26 months. The median OS was 64.0 months and CSS was 135.0 months. Diagnosis year, marital status, histologic type, chemotherapy, T stage, N stage, M stage, tumor size, median household income, and radiation modality were factors influencing the median survival time of OS and CSS. Age was an independent factor influencing OS. The C-index for OS and CSS predictions were 0.72 [95% confidence intervals (CI), 0.70 to 0.74] and 0.73 (95% CI, 0.71 to 0.75), respectively. Calibration plots showed good agreement between nomogram predictions and actual observations.

**Conclusion:**

The nomograms can objectively and precisely predict the OS and CSS of cervical cancer patients receiving radical radiotherapy.

## 1 Introduction

Cervical cancer is the fourth most frequently diagnosed cancer and the fourth leading cause of cancer-related death in women, with an estimated 604,000 new cases and 342,000 deaths worldwide in 2020 (1). For early-stage cervical cancer patients, radical trachelectomy or radical hysterectomy combined with pelvic lymphadenectomy is an important treatment option with long-term tumor control (2–4). Radical chemoradiotherapy is the standard treatment for cervical cancer patients with stage International Federation of Gynecology and Obstetrics (FIGO) IIB and higher. External beam radiotherapy (EBRT) with concurrent cisplatin-based chemotherapy combined with brachytherapy (BT) boost is an important treatment option for patients with locally advanced cervical cancer (5–7). Several studies have shown that patient-related factors, such as age (8–14), histopathology (8, 9, 12, 13, 15–18), stage (8, 11, 12, 14, 17, 19), and tumor size (9, 11, 13, 15, 18), and treatment-technology-related factors, such as chemotherapy (8, 13, 14, 19) or radiotherapy technology (8, 11, 12, 20, 21), had been confirmed as influencing factors for clinical outcomes.

The Surveillance, Epidemiology, and End Results (SEER) program provides information on cancer statistics and is an authoritative source for cancer statistics in the United States. Case ascertainment and data collection for the SEER program started on 1 January 1973, with diagnoses in several geographic areas of the United States and its territories. Geographic area and demographic coverage have expanded over the past 50 years representing approximately 50% of the U.S. population today. Compared with single-institution datasets, the SEER database offers an advantage in clinical research, allowing researchers to access a vast array of cancer cases, explore rare subtypes, analyze long-term outcomes, and uncover broader patterns and trends (11, 14, 22, 23).

Nomograms serve as graphical manifestations of the outcomes derived from the multivariate regression model. Through its user-friendly design, nomograms offer a more intuitive and efficient way to assess various factors and their combined impact on patient outcomes, thus facilitating more informed decision-making in the medical field. While several nomograms have been developed for cervical cancer, they have multiple limitations. They often lack comprehensiveness in terms of patient age range and specific treatment scenarios and primarily focus on surgical cohorts or mixed treatment populations, lacking specificity for radical radiotherapy patients (22–25). Moreover, nomograms may become less accurate over time due to improvements in treatment modalities, changes in the natural history of the disease, advancements in radiotherapy techniques, and evolving treatment paradigms that have occurred over the past two decades (26). Additionally, critical prognostic factors such as socioeconomic status and marital status are often underrepresented in current models. To date, no nomogram based on the SEER database incorporating patients treated with radical radiotherapy across all ages has been reported to predict OS and CSS.

This study addresses this gap by constructing and validating competitive-risk model (CRM) nomograms exclusively for cervical cancer patients treated with radical radiotherapy using the SEER database (2000–2020) to predict OS and CSS probabilities.

## 2 Materials and methods

This study was exempt from the institutional review board and patient consent requirements per U.S. federal regulations (45 CFR 46) due to the use of publicly available, anonymized SEER data. Cervical cancer cases treated with radiotherapy were retrieved from SEER database via SEER*Stat software 8.4.2 [SEER Program^1^ SEER*Stat Database: Incidence—SEER Research Data, 17 Registries, Nov 2022 Sub (2000–2020)—Linked To County Attributes—Time-Dependent (1990–2021) Income/Rurality, 1969–2021 Counties, National Cancer Institute, DCCPS, Surveillance Research Program, released April 2023, based on the November 2022 submission].

The inclusion criteria were as follows: pathological diagnosis of cervical cancer; diagnosis between 2000 and 2020; age at diagnosis between 20 and 79 years. The main exclusion criteria were patients with other malignant tumors, those who had cancer-directed surgery, and patients with incomplete data.

The following variables were extracted from the SEER database: age, year of diagnosis, race, marital status at diagnosis, median household income, histologic type, radiation recodes, chemotherapy recodes, Tumor - Node - Metastasis stage, vital status recodes, cause of death to site recodes, survival months, SEER cause-specific death classification and SEER other cause of death classification. Radiation recodes were categorized into two groups based on SEER coding criteria: (1) EBRT only (beam radiation) and (2) EBRT with BT (combination of beam with implants or isotopes). This classification reflects the standard of care for locally advanced cervical cancer, where BT is typically delivered as a boost to the primary tumor after EBRT. Tumor staging systems differed based on the diagnosis year. For patients from 2000 to 2015, tumor stage refers to the American Joint Committee on Cancer (AJCC) staging system, where different versions correspond to patients in different years (3rd for 2000–2003; 6th for 2004–2015). Tumor stage for patients from 2016 to 2017 refers to SEER combined, and that for patients of 2018 + refers to the extent of disease (EOD) 2018. Tumor histopathology was classified according to the SEER’s implementation of the International Classification of Diseases for Oncology (ICDO), Third Edition.

Statistical analysis was conducted using IBM SPSS 23.0 (IBM, Armonk, NY) and R software (version 4.3.1).^2^ A *p*-value < 0.05 was regarded as significant. OS (event = death from any cause) and CSS (event = death from cervical cancer) curves were plotted by the Kaplan–Meier method. The median survival times were calculated with this method, and group differences were compared using the log rank test.

Patients were randomly assigned to a training set and a validation set at a 4:1 ratio using the createDataPartition function from the caret package in R. The following parameters were used: times = 1, *p* = 0.8, list = FALSE. This ensured a balanced distribution of key variables such as stage (T: *p* = 0.833; N: *p* = 0.747; M: *p* = 0.997) and histology (*p* = 0.806) across the two sets. For the training set, univariate and multivariate Cox regression analyses were performed. Competing risk survival analysis was performed using the crr function (implementing the Fine-Gray model) from the cmprsk package in R. Based on the results of this analysis, the nomograms were created using the nomogram function (rms package) to predict the 3-year and 5-year OS and CSS probabilities, enabling the visualization of these prediction results. Calibration curves with 95% confidence intervals (CIs) were generated using the calibrate function in the rms package with 1,000 bootstrap resamples (B = 1,000) to correct for overfitting. Harrell’s concordance index (C-index) and calibration curves were used to assess the nomograms’ consistency in both sets.

## 3 Results

During the screening process of patients with cervical cancer from SEER between 2000 and 2020 (n = 27,105), several cases were excluded. Specifically, 11,485 cases were excluded due to cancer-directed surgery. Additionally, 954 cases with unknown T stage, 1,280 with unknown N stage, 39 with unknown M stage, 519 with unknown marital status at diagnosis, 45 with unknown race, and 3,973 with unknown tumor size were excluded. After these exclusions, 8,810 patients remained for OS and CSS analysis (Supplementary Figure S1). Among these 8,810 patients, the majority (51.9%) were aged 40–59 years, followed by those aged 60–79 years (27.5%). Squamous cell carcinoma was the most common histology (77.9%), and most patients (89.9%) received chemotherapy. In terms of patient race, the majority were white (72.6%), followed by Black (15.5%). After random grouping, 7,050 patients were assigned to the training set and 1,760 to the validation set. The characteristics are detailed in Table 1.

**Table 1:** Characteristics of the total, training set, and validation set patients.

Characteristic	Total (*n* = 8,810)	Training set (*n* = 7,056)	Validation set (*n* = 1,760)	*p*-value
Age (y), n (%)				0.497
20–39	1,819 (20.6)	1,435 (20.4)	381 (21.8)	
40–59	4,571 (51.9)	3,691 (52.4)	880 (50.0)	
60–79	2,420 (27.5)	1,924 (27.3)	496 (28.2)	
Year of diagnosis n (%)				0.72
2000–2004	1,137 (12.9)	931 (13.2)	206 (11.7)	
2005–2009	1,873 (21.3)	1,502 (21.3)	371 (21.1)	
2010–2014	2,221 (25.2)	1,757 (24.9)	464 (26.4)	
2015–2020	3,579 (40.6)	2,860 (40.6)	719 (40.9)	
Race n (%)				0.935
White	6,400 (72.6)	5,128 (72.7)	1,272 (72.3)	
Black	1,368 (15.5)	1,083 (15.4)	285 (16.2)	
Other	1,042 (11.8)	839 (11.9)	203 (11.5)	
Marital status n (%)				0.352
Married	3,486 (39.6)	2,803 (39.8)	683 (38.8)	
Single	2,983 (33.9)	2,351 (33.3)	632 (35.9)	
Other	2,341 (26.6)	1,896 (26.9)	445 (25.3)	
Histologic Type n (%)				0.806
SCC	6,867 (77.9)	5,476 (77.7)	1,391 (79.0)	
Adenocarcinoma	973 (11.0)	791 (11.2)	182 (10.3)	
Other	970 (11.0)	783 (11.1)	187 (10.6)	
Chemotherapy n (%)				0.634
Yes	7,918 (89.9)	6,347 (90.0)	1,571 (89.3)	
No/unknown	892 (10.1)	703 (10.0)	189 (10.7)	
T stage n (%)				0.833
T0-1	1,776 (20.2)	1,431 (20.3)	345 (19.6)	
T2	3,764 (42.7)	3,021 (42.9)	743 (42.2)	
T3	2,715 (30.8)	2,146 (30.4)	569 (32.3)	
T4	555 (6.3)	452 (6.4)	103 (5.9)	
N stage n (%)				0.747
N0	5,326 (60.5)	4,248 (60.3)	1,078 (61.3)	
N1	3,484 (39.5)	2,802 (39.7)	682 (38.8)	
M stage n (%)				0.997
M0	7,504 (85.2)	6,006 (85.2)	1,498 (85.1)	
M1	1,306 (14.8)	1,044 (14.8)	262 (14.9)	
Tumor size (mm) n (%)				0.905
0–49	2,424 (27.5)	1,935 (27.4)	489 (27.8)	
50–69	3,514 (39.9)	2,794 (39.6)	720 (40.9)	
70–89	2,064 (23.4)	1,662 (23.6)	402 (22.8)	
90+	808 (9.2)	659 (9.3)	149 (8.5)	
Household income ($1,000) n (%)				0.905
0–54	2,424 (27.5)	1,935 (27.4)	489 (27.8)	
55–64	3,514 (39.8)	2,794 (39.6)	720 (40.9)	
65–74	2,064 (23.4)	1,662 (23.6)	402 (22.8)	
75+	808 (9.2)	659 (9.3)	149 (8.5)	
Radiation recodes n (%)				0.909
EBRT only	4,995 (56.7)	3,989 (56.6)	1,006 (57.2)	
EBRT with BT	3,815 (43.3)	3,061 (43.4)	754 (42.8)	

SCC, Squamous cell carcinoma.

With a median follow-up of 26 months (range: 0–251 months), the median OS and CSS of this cohort were 64.0 months (95% CI: 57.8–70.2 months) and 135.0 months (95% CI: not reached), respectively (Supplementary Figure S2).

The log rank test indicated that age, diagnosis year, marital status, histologic type, chemotherapy, T stage, N stage, M stage, median household income, and radiation recodes all influenced the median survival time of OS and CSS. Race was a factor only affecting the median survival time of OS; see Tables 2, 3 and Supplementary Figures S3, S4. In the multivariate analysis of the patients in the training set, the independent factors for both OS and CSS were diagnosis year, marital status, histologic type, chemotherapy, TNM stage, tumor size, median household income, and radiation modality. Age was the independent factor only for OS; see Tables 2, 3.

**Table 2:** Univariate and multivariate Cox regression analysis of factors associated with overall survival.

Variables	Log rank test for total patients			Univariate analysis for training sets		Multivariate analysis for training sets	
	Median survival time (months, 95% CI)	*χ* ^2^	*p*-value	HR (95% CI)	*p*-value	HR (95% CI)	*p*-value
Age (y)		138.9	<0.001		<0.001		<0.001
20–39	188.0 (NR)			Reference		Reference	
40–59	73.0 (61.1–85.0)			1.121 (1.019–1.233)	0.019	0.986 (0.894–1.087)	0.776
60–79	40.0 (35.2–44.8)			1.546 (1.513–1.753)	<0.001	1.276 (1.143–1.425)	<0.001
Year of diagnosis		43.9	<0.001		<0.001		<0.001
2000–2004	65.0 (50.3–79.7)			Reference		Reference	
2005–2009	52.0 (40.2–63.8)			1.084 (0.974–1.206)	0.141	0.909 (0.816–1.014)	0.088
20102014	48.0 (40.0–56.0)			1.163 (1.046–1.293)	0.005	0.864 (0.772–0.966)	0.01
2015–2020	NR (NR)			0.872 (0.781–0.975)	0.016	0.710 (0.632–0.799)	<0.001
Race		25.4	<0.001		<0.001		0.129
White	65.0 (57.8–72.2)			Reference		Reference	
Black	43.0 (33.0–53.0)			1.165 (1.063–1.276)	0.001	1.033 (0.940–1.136)	0.501
Other	93.0 (67.7–118.3)			0.855 (0.763–0.959)	0.007	0.897(0.799–1.009)	0.07
Marital status		82.2	<0.001		<0.001		<0.001
Married	101.0 (84.0–118.0)			Reference		Reference	
Single	54.0 (43.9–64.1)			1.250 (1.150–1.358)	<0.001	1.203 (1.104–1.311)	<0.001
Other	46.0 (33.4–52.6)			1.428 (1.313–1.553)	<0.001	1.302 (1.194–1.419)	<0.001
Histologic type		55.8	<0.001		<0.001		<0.001
Squamous cell carcinoma	70.0 (61.1–78.9)			Reference		Reference	
Adenocarcinoma	69.0 (55.9–82.1)			0.968 (0.864–1.085)	0.575	1.163 (1.036–1.306)	0.011
Other	32.0 (26.7–37.3)			1.394 (1.229–1.506)	<0.001	1.274 (1.149–1.412)	<0.001
Chemotherapy		236.7	<0.001		<0.001		<0.001
Yes	74.0 (65.4–82.6)			Reference		Reference	
No/unknown	20.0 (16.5–23.5)			1.955 (1.772–2.157)	<0.001	1.808 (1.634–2.001)	<0.001
T stage		836.7	<0.001		<0.001		<0.001
T1	173.0 (142.9–203.1)			Reference		Reference	
T2	128.0 (108.7–147.3)			1.111 (1.001–1.234)	0.047	1.116 (1.004–1.241)	0.042
T3	25.0 (22.9–27.1)			2.455 (2.216–2.720)	<0.001	1.916 (1.7718–2.137)	<0.001
T4	17.0 (14.6–19.4)			3.238 (2.808–3.734)	<0.001	1.979 (1.701–2.303)	<0.001
N stage		306.7	<0.001		<0.001		<0.001
N0	111.0 (98.4–123.6)			Reference		Reference	
N1	29.0 (26.3–31.7)			1.737 (1.620–1.862)	<0.001	1.273 (1.174–1.381)	<0.001
M stage		1273.6	<0.001		<0.001		<0.001
M0	101.0 (90.2–111.8)			Reference		Reference	
M1	13.0 (12.0–14.0)			3.515 (3.240–3.814)	<0.001	2.285 (2.079–2.511)	<0.001
Tumor size		329.8	<0.001		<0.001		<0.001
0–49	118.0 (99.3–136.7)			Reference		Reference	
50–69	72.0 (59.7–84.3)			1.221 (1.115–1.337)	<0.001	1.167 (1.064–1.280)	0.001
70–89	38.0 (31.6–44.4)			1.569 (1.423–1.731)	<0.001	1.260 (1.138–1.395)	<0.001
90+	18.0 (15.9–20.1)			2.488 (2.207–2.804)	<0.001	1.505 (1.327–1.708)	<0.001
Median household income		41.7	<0.001		<0.001		<0.001
0–54	54.0 (42.1–65.9)			Reference		Reference	
55–64	46.0 (37.9–54.1)			1.037 (0.934–1.151)	0.495	1.016 (0.914–1.129)	0.771
65–74	65.0 (52.7–77.3)			0.910 (0.824–1.005)	0.063	0.885 (0.800–0.979)	0.018
75+	88.0 (71.1–104.9)			0.788 (0.715–0.868)	<0.001	0.832 (0.751–0.921)	<0.001
Radiation recodes		567.9	<0.001		<0.001		<0.001
EBRT with BT	141.0 (124.6–157.4)			Reference		Reference	
EBRT only	24.0 (23.9–28.1)			2.048 (1.911–2.194)	<0.001	1.528 (1.420–1.644)	<0.001

CI, confidence interval; HR, Hazard ratio; NR, not reached.

**Table 3:** Univariate and multivariate Cox regression analysis of factors associated with cause-specific survival.

Variables	Log rank test for total patients			Univariate analysis for training sets		Multivariate analysis for training sets	
	Median survival time (months, 95% CI)	*χ* ^2^	*p* value	HR (95% CI)	*p* value	HR (95% CI)	*p* value
Age (y)		20.5	<0.001		0.002		0.153
20–39	NR (NR)			Reference		Reference	
40–59	142.0 (NA-NA)			1.065 (0.963–1.177)	0.221	0.916 (0.827–1.016)	0.096
60–79	73.0 (49.5–96.5)			1.208 (1.163–1.367)	0.001	0.976 (0.865–1.101)	0.694
Year of diagnosis		49	<0.001		<0.001		<0.001
2000–2004	176.0 (NR)			Reference		Reference	
2005–2009	91.0 (NR)			1.184 (1.050–1.336)	0.006	0.958 (0.847–1.083)	0.491
2010–2014	78.0 (NR)			1.244 (1.105–1.400)	<0.001	0.886 (0.781–1.004)	0.057
2015–2020	NR (NR)			0.896 (0.792–1.013)	0.08	0.693 (0.609–0.790)	<0.001
Race		20	<0.001		0.001		0.245
White	131.0 (NR)			Reference		Reference	
Black	81.0 (39.6–122.4)			1.131 (1.022–1.251)	0.018	0.999 (0.900–1.110)	0.988
Other	219.0 (NR)			0.847 (0.747–0.960)	0.009	0.897 (0.789–1.019)	0.095
Marital status		43.4	<0.001		<0.001		<0.001
Married	NR (NR)			Reference		Reference	
Single	94.0 (NR)			1.244 (1.137–1.361)	<0.001	1.174 (1.070–1.288)	0.001
Other	83.0 (54.7–111.3)			1.310 (1.194–1.438)	<0.001	1.258 (1.143–1.384)	<0.001
Histologic type		62.1	<0.001		<0.001		<0.001
Squamous cell carcinoma	175.0 (NR)			Reference		Reference	
Adenocarcinoma	105.0 (62.8–147.2)			0.956 (0.844–1.084)	0.483	1.165 (1.026–1.323)	0.018
Other	40.0 (26.8–53.2)			1.411 (1.264–1.575)	<0.001	1.319 (1.180–1.474)	<0.001
Chemotherapy		140.5	<0.001		<0.001		<0.001
Yes	168.0 (NR)			Reference		Reference	
No/unknown	28.0 (20.7–35.3)			1.780 (1.592–1.999)	<0.001	1.707 (1.522–1.915)	<0.001
T stage		811.7	<0.001		<0.001		<0.001
T1	NR (NR)			Reference		Reference	
T2	NR (NR)			1.193 (1.059–1.343)	0.004	1.195 (1.060–1.348)	0.004
T3	29.0 (25.9–32.1)			2.702 (2.406–3.034)	<0.001	2.033 (1.798–2.298)	<0.001
T4	19.0 (16.5–21.5)			3.688 (3.159–4.307)	<0.001	2.147 (1.820–2.532)	<0.001
N stage		386.3	<0.001		<0.001		<0.001
N0	NR (NR)			Reference		Reference	
N1	34.0 (30.1–37.9)			1.952 (1.809–2.105)	<0.001	1.356 (1.242–1.480)	<0.001
M stage		1,346	<0.001		<0.001		<0.001
M0	NR (NR)			Reference		Reference	
M1	14.0 (12.9–15.1)			3.903 (3.582–4.253)	<0.001	2.437 (2.206–2.692)	<0.001
Tumor size		400.6	<0.001		<0.001		<0.001
0–49	NR (NR)			Reference		Reference	
50–69	154.0 (NR)			1.367 (1.233–1.516)	<0.001	1.252 (1.128–1.390)	<0.001
70–89	52.0 (34.8–69.2)			1.821 (1.632–2.033)	<0.001	1.376 (1.228–1.542)	<0.001
90+	20.0 (17.0–23.0)			2.997 (2.631–3.414)	<0.001	1.704 (1.485–1.955)	<0.001
Median household income		35.4	<0.001		<0.001		0.01
0–54	142.0 (NR)			Reference		Reference	
55–64	67.0 (42.0–92.0)			1.105 (0.985–1.240)	0.09	1.061 (0.944–1.191)	0.321
65–74	128.0 (NR)			0.961 (0.858–1.070)	0.444	0.925 (0.827–1.035)	0.175
75+	210.0 (NR)			0.863 (0.748–0.929)	0.001	0.894 (0.799–1.001)	0.051
Radiation recodes		543.9	<0.001		<0.001		<0.001
EBRT with BT	NR (NR)			Reference		Reference	
EBRT only	33.0 (29.4–36.6)			2.160 (2.002–2.331)	<0.001	1.570 (1.449–1.702)	<0.001

CI, confidence interval; HR, Hazard ratio; NR, Not reached.

**Figure 1:**
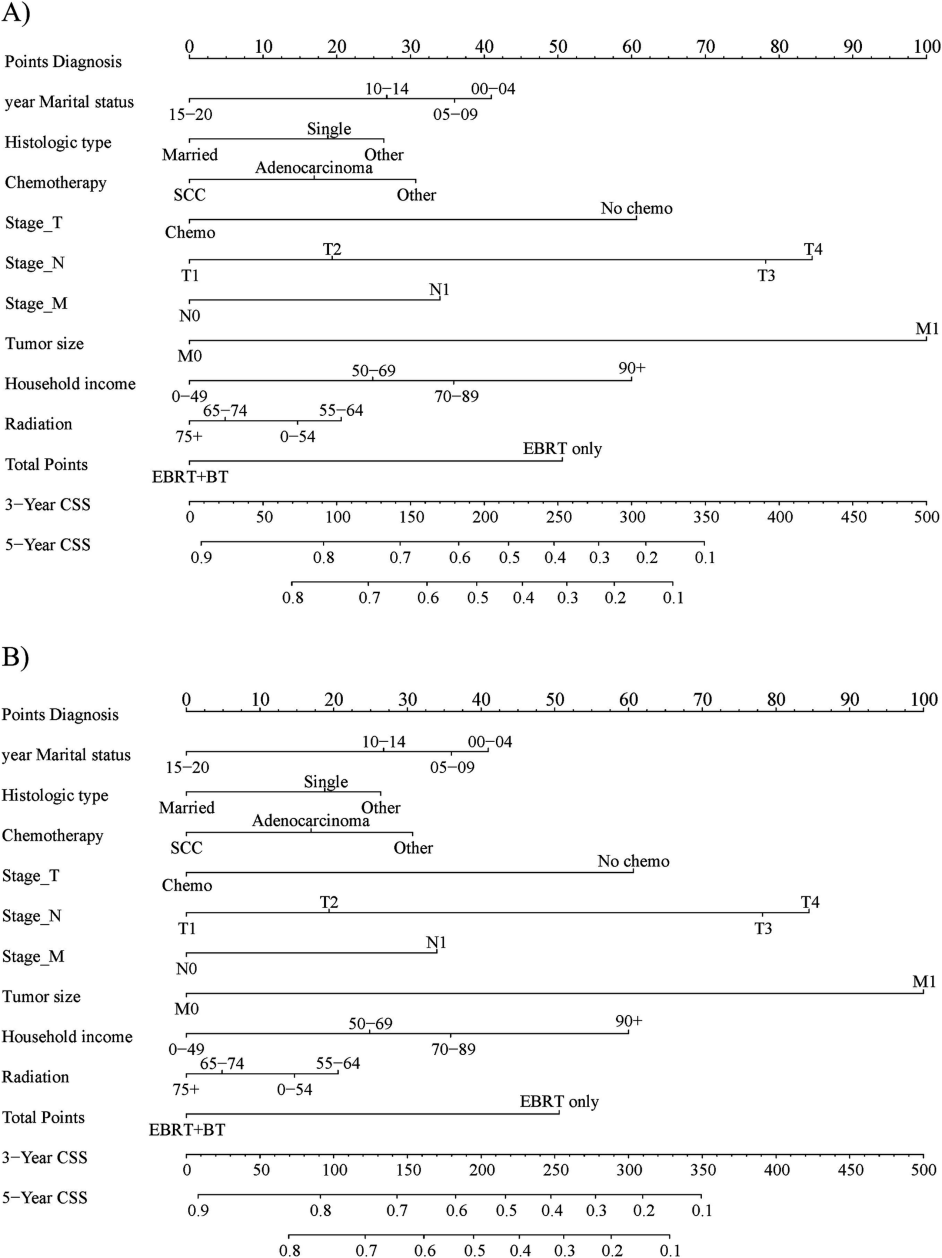
The prognostic nomogram for 3-year and 5-year **(A)** overall survival and **(B)** cause-specific survival in the training set patients.

**Figure 2:**
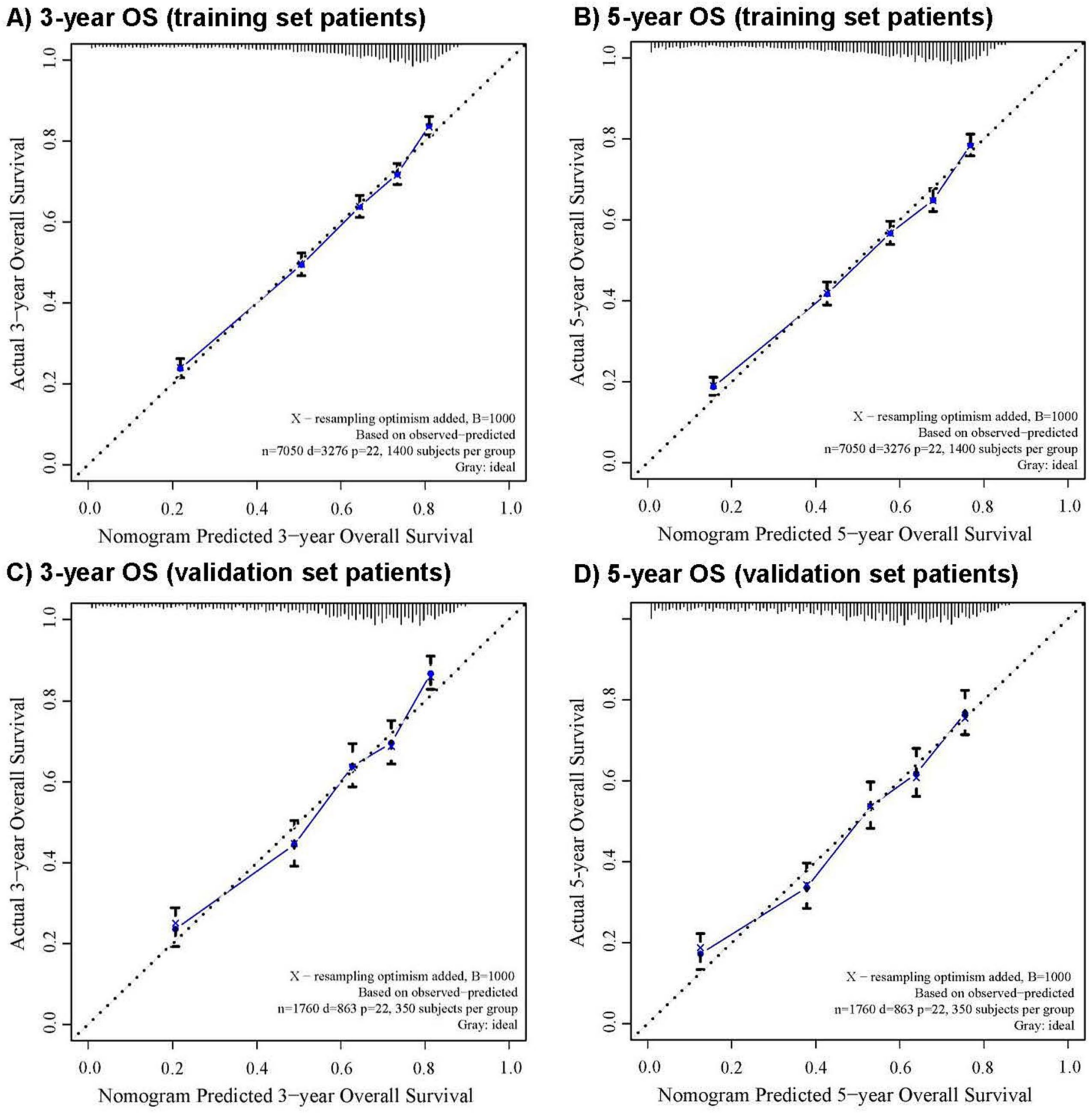
The calibration curve for predicting overall survival with 95% confidence intervals calculated via 1,000 bootstrap resamples at **(A)** 3-year and **(B)** 5-year in the training set patients, and at **(C)** 3-year and **(D)** 5-year in the validation set patients. Nomogram predicted probability of overall survival is plotted on the x-axis; actual overall survival is plotted on the y-axis.

**Figure 3:**
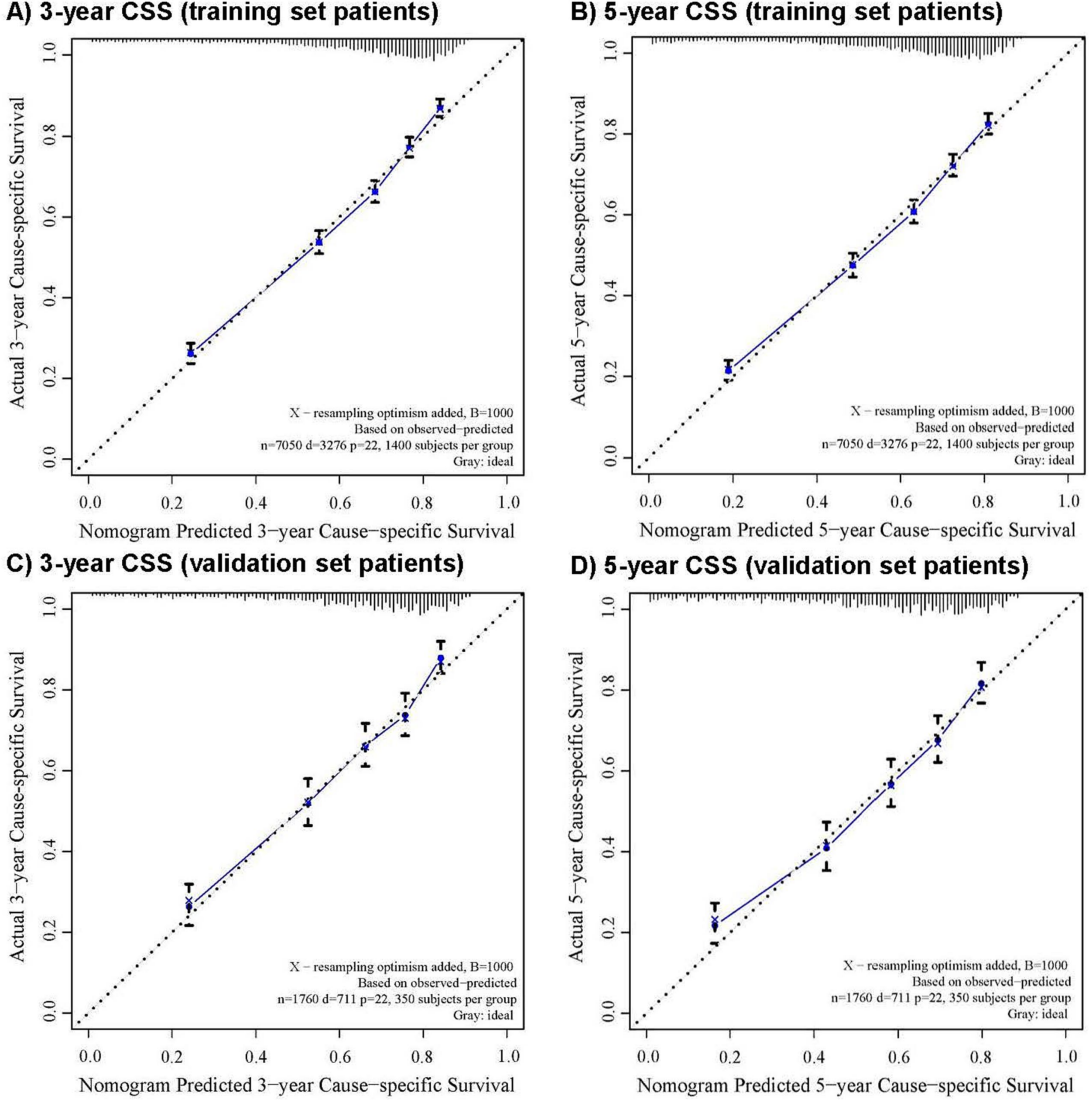
The calibration curve for predicting cause-specific survival with 95% confidence intervals calculated via 1,000 bootstrap resamples at **(A)** 3-year and **(B)** 5-year in the training set patients, and at **(C)** 3-year and **(D)** 5-year in the validation set patients. Nomogram predicted probability of cause-specific survival is plotted on the x-axis; actual cause-specific survival is plotted on the y-axis.

The CRM nomograms integrating all significant independent factors for OS and CSS in patients in the training set is shown in Figures 1A,B, respectively. The C-index for OS and CSS predictions were 0.72 (95% CI: 0.70 to 0.74) and 0.73 (95% CI, 0.71 to 0.75), respectively. Calibration plots showed good agreement between nomogram predictions and actual observations, with 95% CI bands closely aligning with the ideal 45-degree line (Figures 2, 3).

## 4 Discussion

This study revealed that there are 11 factors influencing OS in cervical cancer patients undergoing radical radiotherapy, while 10 factors affect CSS. These factors can be categorized into three groups: 1. patient-related factors, including age (affecting only OS), diagnosis year, marital status, and median household income; 2. tumor-related factors, such as histologic type, TNM stage, and tumor size; and 3. treatment technology-related factors, such as chemotherapy and radiation therapy modality.

Our research indicated that age is a factor influencing OS. Compared with patients aged 20–39 years, those aged 60–79 years had a significantly decreased OS, with a hazard ratio (HR) of 1.276 (1.143–1.425), *p* < 0.001. Similarly, the research results of Hou et al. suggested that patients younger than 70 years had better OS than elderly patients, while the CSS was comparable between the two age groups (12). The higher number of non-cancer-related deaths among elderly patients might be the main reason for the poorer OS in the elderly group. Thus, if an elderly patient’s physical condition can tolerate treatment, active treatment should be provided.

Diagnosis year was included as a predictor due to its strong association with the progressive improvement in radiotherapy and systemic therapies over time. Between 2005 and 2012, the European Group of Curietherapie-European Society for Therapeutic Radiology and Oncology (GEC-ESTRO) established the basic concepts and terminology of three-dimensional image-guided BT (3D-IGBT) for cervical cancer and published a series of recommendations for its implementing. In 2016, the International Commission on Radiation Units and Measurements (ICRU) published Report No. 89, laying the foundation for image-guided adaptive BT for cervical cancer, namely 4D-IGABT. The European study on MRI-guided BT in locally advanced cervical cancer (EMBRACE), which commenced in 2008 and was completed in 2015, along with a series of dose–response studies on radical radiotherapy for cervical cancer, established the dose constraints for target volumes and organs at risk, further guiding the clinical practice of radical radiotherapy for cervical cancer (27–29). The adoption of 3D-IGBT (2005+) and 4D-IGABT (2016+) significantly improved local control rates, while the introduction of immunotherapy (2018+) and targeted agents (2014+) expanded treatment options. These innovations likely drove improvements in survival. Another reason why the diagnosis year was included as one of the predictive factors in the nomogram is that it is an independent prognostic factor in the multivariate analysis.

The implementation of 3D-IGBT and IGABT and the application of combined intracavitary and interstitial BT for patients with large residual tumors and poor responses after EBRT have effectively enhanced the efficacy of radical radiotherapy for cervical cancer (5, 6, 30–32). Although radiation recodes are also one of the factors affecting OS and CSS in this study, due to the limitations of the SEER database, only EBRT alone and EBRT combined with BT can be distinguished. Moreover, in the past two decades, adjuvant treatment methods that have gradually emerged, such as immunotherapy and targeted therapy, have also improved the OS and progression-free survival (PFS) of cervical cancer patients (33, 34). Thus, advancements in the BT field, immunotherapy, and targeted therapy are not reflected in the radiation records factor but might manifest in the year of diagnosis.

In addition to the impact of age on survival, marital status also emerged as a significant factor influencing both OS and CSS in our study. This finding has been corroborated by previous research. In the prediction model developed by Jiang et al., marital status was identified as a factor affecting OS and CSS in cervical cancer patients during univariate analysis, with the single group showing inferior outcomes compared to the married group. In multivariate analysis, marital status remained an independent prognostic factor solely for CSS, again with the single group faring worse than the married group (22). Median household income also served as an independent prognostic factor for OS and CSS in our patient cohort. Similarly, research by Zreik et al. indicated that lower household income was linked to poorer OS for stages I, III, and IV diseases (35).

Multiple studies have shown that locally advanced cervical cancer patients with adenocarcinoma (AC) histology have significantly worse survival outcomes compared to those with squamous cell carcinoma (SCC). AC has been proven to be an independent prognostic factor associated with poorer OS (9, 16, 18). The low radio-sensitivity of cervical AC contributes to its worse survival prognosis. Given the lower survival rate of patients with cervical AC, it is necessary to develop more effective strategies for them. The application of new cytotoxic drugs such as paclitaxel is one approach to improve the survival rate of AC patients (36). Another effective method for improving the survival rate of AC patients is the utilization of modern radiation therapy modalities, such as IGABT (5, 30, 32). Based on the dose–effect relationship of radical radiotherapy for cervical cancer, higher doses delivered to primary tumors are associated with greater tumor control (27, 28, 31). Thus, for cervical AC patients with low radio-sensitivity, when the doses to OARs are within the acceptable dose constraints, higher doses may be beneficial for their survival.

In the radical radiotherapy of cervical cancer, TNM stage and tumor size have long been regarded as independent prognostic factors for the survival of cervical cancer patients (8, 11–13, 19). In the dose–response relationship study conducted by Tanderup et al., probit models were developed for stage II and III + IV subgroups (29). The findings indicated that the dose–response curve of stage III + IV patients was significantly lower than that of stage II patients. In the dose range of high-risk clinical target volume (HR-CTV) D90 80-90Gy_EQD2_, compared with the predicted 3-year local control rate for stage II patients, stage III + IV patients was 10–17% lower.

A larger tumor size at the time of diagnosis may result in a larger residual tumor volume after EBRT, thereby posing greater challenges in BT (6). Pötter et al. revealed that patients with tumor sizes of 2–5 cm had significantly higher CSS and local control (LC) than those with tumor sizes greater than 5 cm, regardless of whether it was between 1998 and 2000 or 2001 and 2003 (37). A study by Ke et al. found that the residual gross tumor volume was an independent factor for 2-year OS, progression-free survival (PFS), and LC, with HRs of 1.05 (1.03–1.07), 1.03 (1.01–1.05), and 1.06 (1.03–1.09), respectively (38). EMBRACE II study showed that during BT, small HR-CTV (<30 cc) were more likely to receive high-dose treatment and achieve high local control, whereas larger HR-CTV (>30 cc) had lower local control rates even when receiving the same high-dose treatment (6). Given that larger tumor sizes pose challenges in brachytherapy, clinicians may consider more aggressive treatment strategies, such as adjusting the radiation dose and fractionation scheme based on tumor size to optimize treatment outcomes.

Concurrent chemotherapy during EBRT has emerged as a critical element in the treatment of locally advanced cervical cancer. Despite the SEER database’s incomplete recording of chemotherapy types, drugs, and doses, this study reaffirmed the significance of chemotherapy in treating cervical cancer. Current evidence has not yet established the definitive role of adjuvant chemotherapy in treating cervical cancer (39, 40). Consequently, neoadjuvant chemotherapy is not recommended for use outside of clinical trials.

Research by Han et al. and Gill et al. demonstrated that BT was linked to higher CSS and OS, highlighting BT as a vital treatment component for locally advanced cervical cancer (20, 21). Our study confirmed these findings once again. Nevertheless, there has been a worrying decline in BT utilization attributed to the increasing adoption of highly conformal radiation therapy techniques, such as intensity-modulated radiotherapy (IMRT) and stereotactic body radiation therapy (SBRT) (21). The BT utilization rate has been on a downward trend since 1988, dropping from 83% in 1988 to 58% in 2009, with a significant plunge to 43% in 2003. Currently, it is a widely accepted consensus that EBRT with concurrent chemotherapy combined with BT is the standard treatment modality for radical radiotherapy of locally advanced cervical cancer patients. The National Comprehensive Cancer Network guidelines for cervical cancer emphasize that BT is an essential part of definitive therapy for all primary cervical cancer patients who are ineligible for surgery (41). Moreover, the guidelines state that conformal EBRT, such as IMRT or SBRT, should not be routinely used as an alternative to BT for treating central disease in patients with an intact cervix.

In nomogram evaluation, the C-index serves as a key metric for assessing a model’s predictive ability. In general, if the C-index reaches or exceeds 0.7, the model is generally considered to have good predictive ability and can aid decision-making to some extent. The C-index values for OS (0.72) and CSS (0.73) in our study are comparable to or exceed those of prior cervical cancer nomograms. For example, Ding et al. (25) reported a C-index of 0.714 for OS and 0.683 for CSS in a SEER-based model including mixed treatment modalities, while Jiao et al. (23) achieved 0.641 for CSS in elderly patients. Our model’s performance is notable given its exclusive focus on radical radiotherapy, a subgroup often underrepresented in previous studies. However, it is important to note that SEER-based models reported higher C-indexes (0.824–0.844), likely due to richer prognostic variables (e.g., metastasis numbers and lymph vascular space invasion) (22). Despite these limitations, our nomogram’s moderate-to-good discrimination supports its clinical utility for risk stratification in routine practice.

The nomograms can be integrated into clinical practice through a three-step framework. First, in the prognostic assessment, physicians input patient characteristics such as age, TNM stage, and radiation modality into the nomogram to calculate 3-year and 5-year OS/CSS probabilities. Then, these probabilities are used in shared decision-making, facilitating patient–physician discussions about treatment goals. They help balance curative intent with potential toxicities; for example, physicians can counsel elderly patients on competing risks of treatment-related morbidity versus non-cancer mortality. Finally, in treatment optimization, high-risk patients (such as those with predicted 5-year CSS < 50%) may benefit from intensified treatment protocols such as dose-escalated brachytherapy, while low-risk patients could avoid overtreatment. This approach aligns with NCCN guidelines that emphasize personalized radiotherapy planning for cervical cancer (41).

The nomogram’s reliance on basic clinical variables (e.g., age, TNM stage, and radiation modality) and its three-step clinical framework (prognostic assessment, shared decision-making, and treatment optimization) make it highly translatable to resource-limited regions. For example, in regions where advanced imaging (e.g., MRI) is scarce, the nomogram can still provide actionable insights using SEER-compatible variables. In low-income countries, the tool could guide triage by identifying patients who may benefit from dose-escalated radiotherapy.

Our nomogram complements NCCN recommendations by enhancing risk stratification through identifying high-risk subgroups such as unmarried patients with low income who may require aggressive treatment despite similar clinical stages, guiding treatment optimization for locally advanced disease by flagging candidates for dose-escalated brachytherapy (e.g., predicted 5-year CSS < 50%) in alignment with NCCN’s emphasis on personalized radiotherapy, and facilitating shared decision-making by quantifying competing risks such as treatment-related morbidity versus non-cancer mortality in elderly patients to balance curative intent with quality of life—a gap in current guidelines.

The SEER database’s lack of detailed treatment information introduces several limitations. First, the use of AJCC staging (instead of FIGO staging) may reduce the external validity of our model, as FIGO staging is more widely adopted in clinical practice and incorporates prognostic factors such as parametrial involvement. Second, chemotherapy type and dose data are not available in SEER, limiting our ability to evaluate the impact of specific regimens (e.g., cisplatin vs. paclitaxel-based chemotherapy) on survival outcomes. Third, radiation dose, fractionation, total treatment time, and tumor size after EBRT are absent, precluding dose–response analyses critical for optimizing radiotherapy protocols. However, our binary classification captures the essential distinction between definitive EBRT with BT (standard of care) and alternative approaches (EBRT only). These limitations highlight the need for future studies to integrate SEER data with institutional records or national registries (e.g., NCDB) to capture missing variables and improve model precision. Previous studies have utilized the SEER database to explore the clinical and non-clinical characteristics that might influence the prognosis of cervical cancer patients and developed nomograms for OS and CSS (22). In contrast, this study concentrated on patients who received radical radiotherapy and had not undergone cancer-directed surgery, with the patient cohort spanning from 2000 to 2020. Moreover, another limitation is that the accuracy of the nomogram results has not been externally validated.

## 5 Conclusion

The nomograms put forward in this study can objectively and precisely predict the OS and CSS of patients with cervical cancer who receive radical radiotherapy. While the current study provides robust internal validation, external validation using independent datasets (e.g., institutional cohorts or the National Cancer Database) is critical to confirm the nomograms’ generalizability. Additionally, efforts should be made to incorporate missing data elements, such as FIGO stage and detailed treatment information, to improve the accuracy and generalizability of the nomograms. Long-term follow-up studies are also needed to better understand the long-term survival trends and the impact of emerging treatment modalities on cervical cancer patients undergoing radical radiotherapy.

## Data Availability

The original contributions presented in the study are included in the article/Supplementary material, further inquiries can be directed to the corresponding author.

## Footnotes

^1^ www.seer.cancer.gov

^2^ http://www.r-project.org
