# Supplementary material for: Construction and validation of nomograms for predicting overall survival and cause-specific survival in cervical cancer patients undergoing radical radiotherapy based on the SEER database
Source: Front Med (Lausanne). 2025 Apr 29;12:1587465. doi: 10.3389/fmed.2025.1587465 (PMC12069064; doi:10.3389/fmed.2025.1587465)
Supplement: Supplementary file 1 [file Supplementary_file_1.docx]

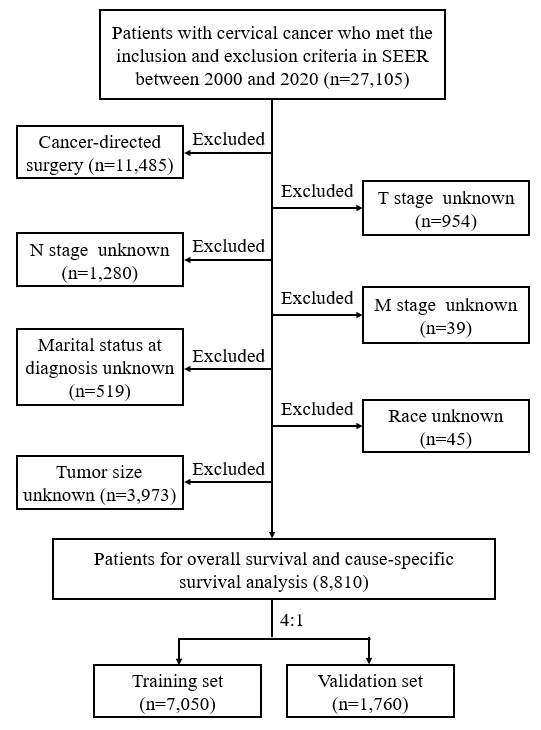


**Fig. S1** The flowchart of study population selection.

**
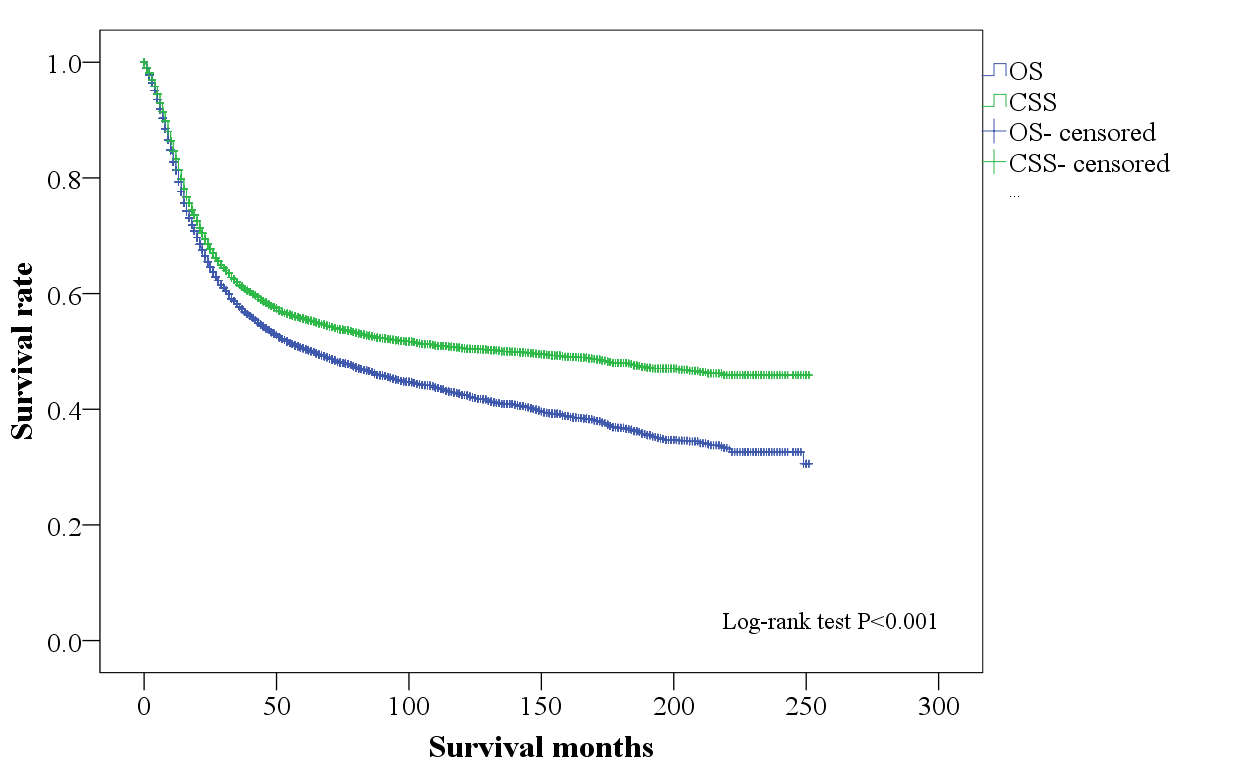
**

**Fig. S2.** Kaplan-Meier survival curve of the included patients screened from SEER database.


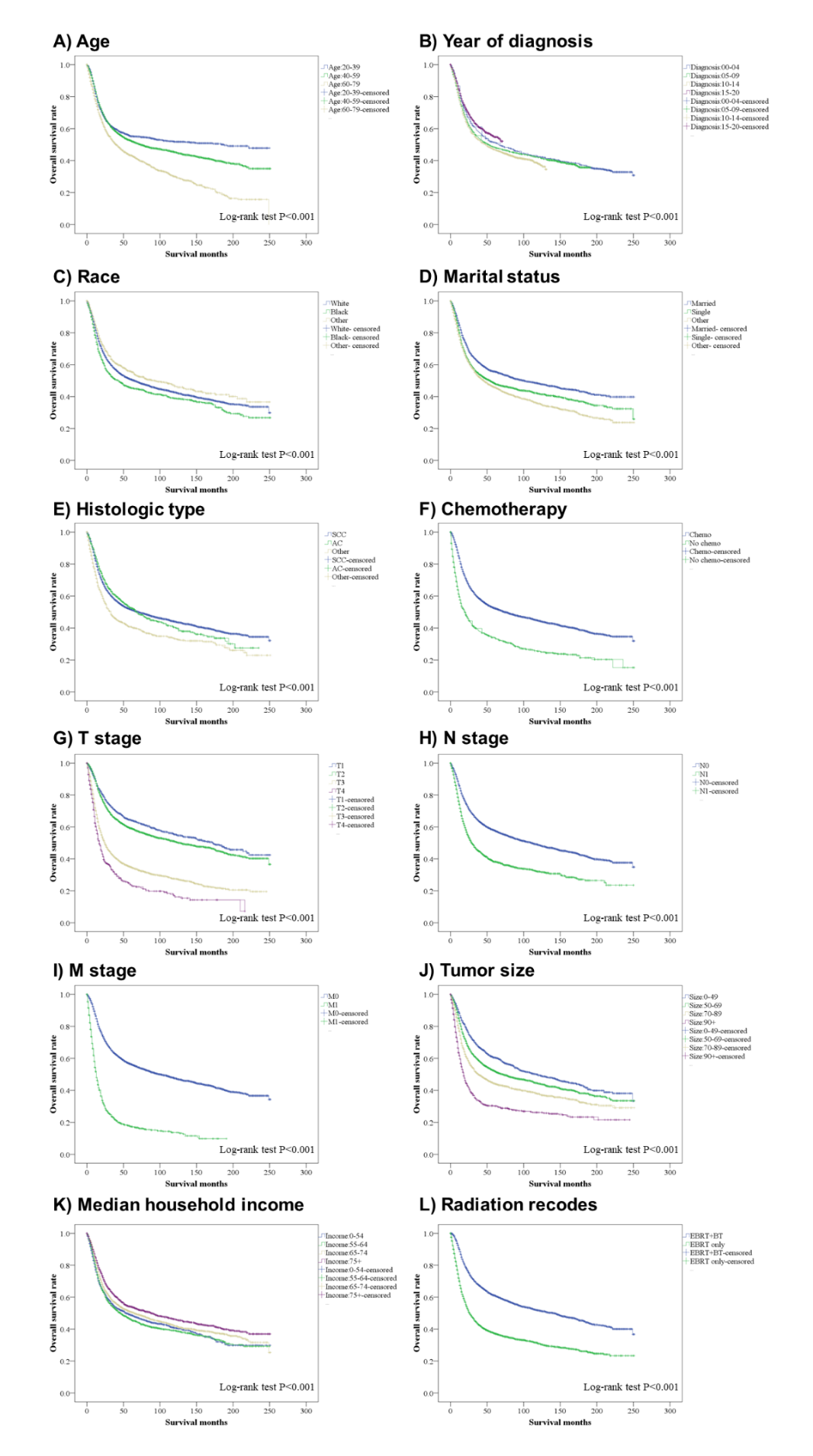


**Fig. S3.** Kaplan-Meier overall survival curve for subgroups according to A) age, B) year of diagnosis, C) race, D) marital status, E) histologic type, F) chemotherapy, G) T stage, H) N stage, I) M stage, J) tumor size, K) median household income and L) radiation recodes.


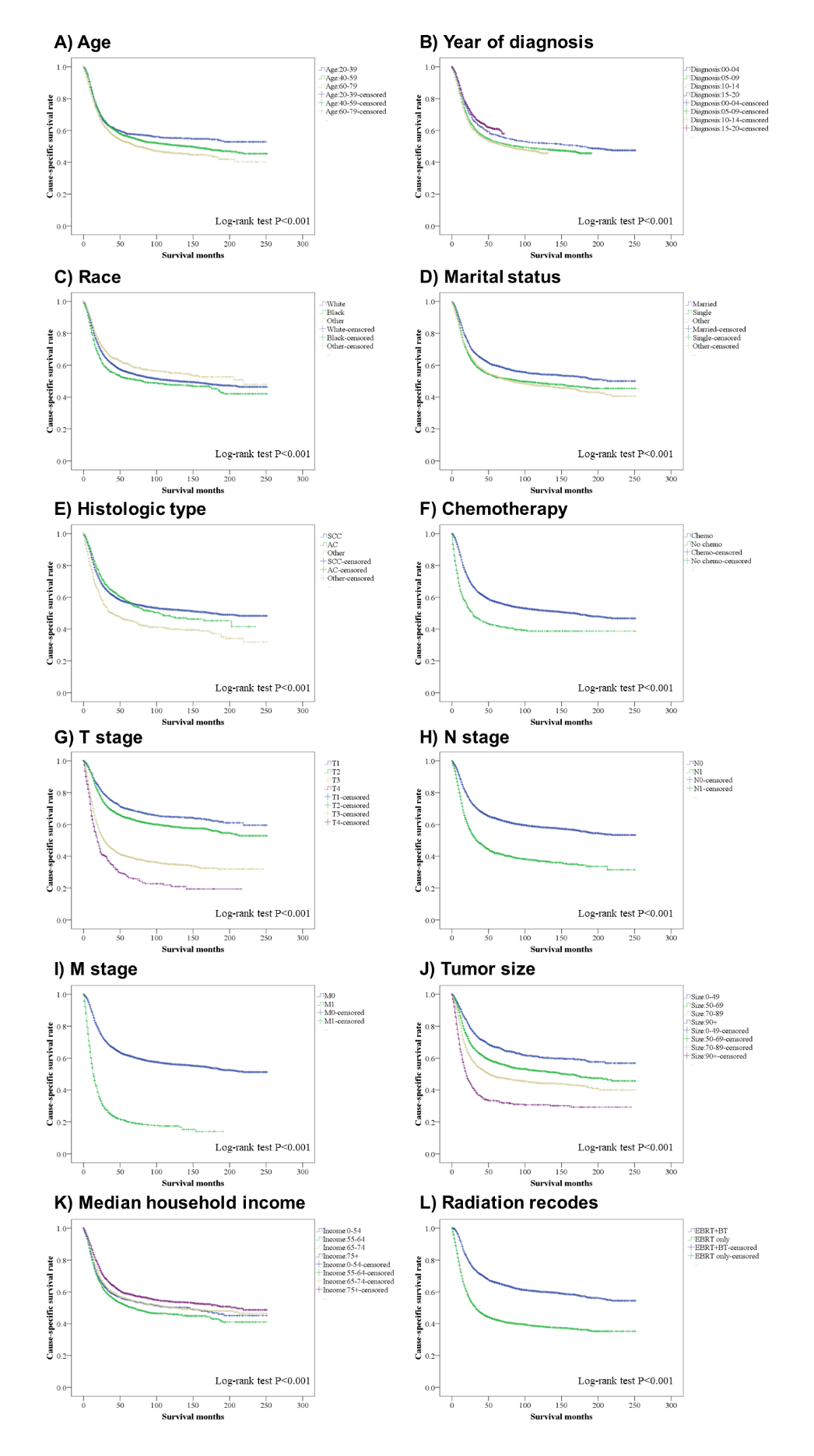


**Fig. S4.** Kaplan-Meier cause-specific survival curve for subgroups according to A) age, B) year of diagnosis, C) race, D) marital status, E) histologic type, F) chemotherapy, G) T stage, H) N stage, I) M stage, J) tumor size, K) median household income and L) radiation recodes.
